# Diagnostic biomarkers for differentiating AQP4-IgG- negative NMOSD from other nervous system autoimmune disorders: a retrospective study

**DOI:** 10.3389/fimmu.2025.1637613

**Published:** 2026-01-14

**Authors:** Wencan Jiang, Yang Jiao, Menglue Zhang, Chenxu Wang, Xiaotong Li, Kelin Chen, Yaowei Ding, Haoran Li, Lijuan Wang, Siwen Li, Ziwei Liu, Chi Huang, Lei Liu, Guojun Zhang

**Affiliations:** 1 Laboratory Diagnosis Center, Beijing Tiantan Hospital, Capital Medical University, Beijing, China; 2 Beijing Engineering Research Center of Immunological Reagents Clinical Research, Beijing, China; 3 NMPA Key Laboratory for Quality Control of In Vitro Diagnostic, Beijing, China; 4 Laboratory Diagnosis Platform for Nervous System Infectious Diseases, Laboratory for Clinical Medicine, Capital Medical University, Beijing, China; 5 Department of Biochemistry and Molecular Biology, School of Basic Medicine, Capital Medical University, Youanmen, Beijing, China

**Keywords:** AQP4-IgG, clinical characteristics, laboratory examinations, magnetic resonance imaging, neuromyelitis optica spectrum disorder

## Abstract

**Background and objective:**

The clinical manifestations of neuromyelitis optica spectrum disorders (NMOSD) overlap with those of other central nervous system (CNS) disorders, making differential diagnosis based on symptoms alone difficult. This study aimed to identify distinguishing indicators of aquaporin-4 (AQP4) IgG-negative NMOSD and assess their diagnostic value.

**Methods:**

We analyzed four groups: 85 patients with AQP4-IgG-negative NMOSD, 192 with AQP4-IgG-positive NMOSD, 547 with other nervous system autoimmune disorders (e.g., multiple sclerosis, Guillain-Barré syndrome, and viral and autoimmune encephalitis), and 269 healthy controls matched for sex, age, and BMI.A diagnostic model was established using clinical, biochemical, and cerebrospinal fluid (CSF) variables. The dataset was divided into training and internal validation cohorts, and model performance was further assessed through external validation using an independently collected dataset obtained during the same study period.

**Results:**

We enrolled 1,093 participants. The AQP4-IgG-negative NMOSD group showed: higher diplopia incidence (35.29% vs. 17.19% in positive NMOSD, 17.18% in controls; P<0.001); lower limb sensory abnormalities (76.47% vs. 89.58%, P = 0.0056) and urinary dysfunction (36.47% vs. 57.29%, P = 0.0017) than positive cases; predominant brainstem involvement (50.59% vs. 31.77% in positive, 28.70% in controls; P<0.001); moderate spinal cord involvement (70.59% vs. 88.02% in positive, 38.57% in controls; P<0.001); and altered thyroxine, apolipoprotein A1, eosinophils, and basophils. After false discovery rate correction (q<0.1), diplopia, brainstem involvement, spinal cord involvement, serum albumin, and CSF albumin remained significant. A combined model incorporating twelve predictors (Ocular symptoms, Gastrointestinal symptoms, Water swallowing and choking difficulties, Cerebral lobes, Centrum semiovale, Brainstem, Cerebral ganglia, Spinal cord, APOA1, Ca, Toxoplasma gondii IgM Antibody, CSF IgG) achieved an AUC of 0.936in the training cohort and 0.929 in external validation.

**Discussion:**

AQP4-IgG-negative NMOSD shows distinct clinical, imaging, and laboratory profiles compared to other nervous system autoimmune disorders. A multi-indicator diagnostic approach offers higher accuracy than single-marker diagnostics.

## 1 Introduction

Neuromyelitis optica spectrum disorder (NMOSD) is a group of autoimmune-mediated inflammatory demyelinating diseases of the central nervous system (CNS) that primarily affect the optic nerve and spinal cord. NMOSD is prevalent in Asian populations and is associated with high rates of recurrence and disability (1, 2). It predominantly affects middle-aged women; however, children and older individuals are not entirely immune, with female-to-male ratios as high as 9–11 (3, 4). Furthermore, NMOSD typically has a relapsing course, with studies showing that approximately 60% and 90% of patients experience relapse within 1 and 3 years, respectively (1, 5, 6).

Aquaporin-4 (AQP4) IgG are highly specific diagnostic biomarkers for NMOSD and play a crucial role in distinguishing NMOSD from other nervous system autoimmune disorders, including multiple sclerosis (MS). The detection rate of AQP4-IgG has gradually increased with continuous improvements in detection technologies (7, 8). However, AQP4-IgG cannot be detected in some cases (9, 10). This may be due to the presence of AQP4-IgG in patients during the window period or the insufficient sensitivity of current detection methods. Additionally, patients with AQP4-IgG-negative NMOSD experience different pathogenic mechanisms than those with AQP4-IgG-positive NMOSD, in which pathogenic antibodies may target other antigens in the body (11). Variations in the AQP4-IgG peptide sequence may lead to the production of different AQP4-IgG subtypes.

NMOSD diagnosis mainly relies on the detection of AQP4-IgG, combined with clinical symptoms and imaging findings, to facilitate differentiation from diseases, including MS (12). However, imaging changes take time to become visibly apparent in patients with AQP4-IgG-negative NMOSD, by which time the disease may have progressed significantly, hindering subsequent treatment. The clinical manifestations of this disease are similar to those of other nervous system autoimmune disorders, making differential diagnosis based solely on clinical symptoms extremely challenging. Therefore, AQP4-IgG-negative NMOSD presents several challenges. In this study, we conducted an in-depth analysis of these diseases using clinical, imaging, and laboratory parameters.

## 2 Materials and method

### 2.1 Patients

Between 2015 and 2024, 85 patients were clinically diagnosed with AQP4-IgG-negative NMOSD at Beijing Tiantan Hospital, affiliated with Capital Medical University. These patients were assigned to the AQP4-IgG–negative NMOSD group. All included cases were either confirmed negative for MOG-IgG or, in a small number of patients, MOG-IgG testing was not performed but MOGAD was clinically excluded. Diagnoses were made according to the 2015 International Panel for NMO Diagnosis (IPND) criteria (13). Inclusion criteria were: (1) age 3–85 years (consistent with cohort age range); (2) clinical manifestations suggestive of NMOSD (optic neuritis, acute myelitis, or brainstem syndrome); (3) follow-up duration ≥12 months; (4) AQP4-IgG negativity confirmed by CBA (detected via KingMed and Newterrain platforms); (5) MOG-IgG negative (53/85 patients, tested by CBA) or MOGAD clinically excluded (32/85 patients, based on absence of MOGAD-typical features: bilateral optic neuritis, pediatric onset). Exclusion criteria were: (1) MS (per 2017 McDonald criteria); (2) MOGAD (MOG-IgG positive by CBA); (3) infectious encephalomyelitis (e.g., bacterial meningitis); (4) non-autoimmune CNS disorders (e.g., cerebrovascular disease); (5) incomplete clinical/imaging data. All clinical, imaging, and laboratory data were collected at the time of initial diagnosis (before immunotherapy initiation) via retrospective review of electronic medical records. Additionally, 192 patients diagnosed with AQP4-IgG-positive NMOSD were included in the AQP4-IgG-positive NMOSD group (13). Moreover, 547 patients diagnosed with other nervous system autoimmune disorders, including 240 cases of MS, 212 cases of Guillain–Barré syndrome (GBS), 85 cases of viral encephalitis (VE), and 10 cases of autoimmune encephalitis (AE), were included in the other nervous system autoimmune disorders control group, following relevant literature guidelines (14–17). Furthermore, a healthy control group of 269 individuals was selected to match the sex ratio, age distribution, and body mass index (BMI) of the AQP4-IgG-negative NMOSD group (P > 0.05).

This study was approved by the Ethics Committee of Tiantan Hospital of Capital Medical University (Approval No. KY2023-157-03).

### 2.2 Research methodology

#### 2.2.1 Clinical information collection

General information was collected for four groups, including gender, age, marital status, education level, birthplace, blood pressure, BMI, heart rate, and presence of underlying conditions (such as hypertension, diabetes, cardiovascular disease, and cerebrovascular disease). The initial symptoms, clinical manifestations, and magnetic resonance imaging (MRI) data (including brain MRI and spinal cord MRI) of patients from the AQP4-IgG-negative NMOSD group, AQP4-IgG-positive NMOSD group, and the other CNS disease control group were recorded and analyzed.

To control for the potential inflation of type I error due to multiple comparisons, we applied the Benjamini–Hochberg false discovery rate (FDR) correction exclusively to univariate analyses (for variable screening, not model selection). The q < 0.1 cutoff was selected for exploratory biomarker identification in this rare disease, as it balances false positive control and retention of potentially biologically relevant variables—strict q < 0.05 might exclude meaningful indicators given the limited sample size of AQP4-IgG-negative NMOSD. Statistical significance was defined as an FDR-adjusted P < 0.1.

#### 2.2.2 Clinical and imaging indicators classification

Clinical manifestations were categorized according to the NMOSD diagnostic criteria established by the international diagnostic panel into 6 groups of core clinical syndromes (optic neuritis, acute myelitis, area postrema syndrome, acute brainstem syndrome, acute diencephalic syndrome, and cerebral syndrome).

In order to enhance the precision and granularity of the analysis, the initial categorization of six clinical symptoms was refined into sixteen more specific and targeted clinical manifestations, corresponding to each of the aforementioned core categories: ocular symptoms (corresponding to optic neuritis), abnormal limb sensation, limb weakness, urinary and fecal incontinence (corresponding to acute myelitis), gastrointestinal symptoms (corresponding to area postrema syndrome), dizziness, diplopia, dysphagia and choking on liquids, facial sensory disturbances, ear symptoms (corresponding to acute brainstem syndrome), hypersomnia, fever (corresponding to acute diencephalic syndrome), headache, language impairment, decreased consciousness level, seizures (corresponding to cerebral syndrome).

These patients were assigned to the AQP4-IgG–negative NMOSD group. All included cases were either confirmed negative for MOG-IgG or, in a small number of patients, MOG-IgG testing was not performed but MOGAD was clinically excluded. Diagnoses were made according to the 2015 International Panel for NMO Diagnosis (IPND) criteria.

For other central nervous system diseases, their clinical features are not entirely identical to NMOSD, but their clinical manifestations can be categorized into the aforementioned 16 clinical manifestations. Therefore, the same classification framework was employed during the analysis and discussion to ensure the systematic and consistent nature of the study. In the analysis of magnetic resonance imaging (MRI) data, after referring to relevant literature, Based on brain anatomical structures, the regions affected by MRI lesions corresponding to the six core clinical symptoms were classified into 10 regions (lobes, ventricles, centrum semiovale, corona radiata, internal capsule, brainstem, cerebellum, thalamus, basal ganglia, and corpus callosum). Special attention was also given to key regions of the cervical and thoracic spinal cord in the spinal cord MRI analysis.

To control for the potential inflation of type I error due to multiple comparisons, we applied the Benjamini–Hochberg false discovery rate (FDR) correction. Statistical significance was defined as an FDR-adjusted P < 0.1.

#### 2.2.3 Instruments and reagents

Using the Mindray BC-6900 fully automated hematology analyzer and its reagents, blood routine related indicators (Neu, Lym, plt, SD) were tested; the Mindray EH2080C fully automated urine analyzer was used to test urine routine related indicators (urine specific gravity, urine pH); indirect immunofluorescence assays employing Euroimmun reagents from Germany were used to detect autoantibodies; Beckman DXI800 fully automated chemiluminescence analyzer was used to measure thyroid function indicators (T3, T4, FT3, FT4, TSH); Hitachi LABOSPECT008 fully automated biochemical analyzer was used to measure biochemical parameters (CK, IBIL, DBIL, UA, Alb); Roche e602 fully automated immunoassay analyzer was used to detect EB virus antibodies and herpes simplex virus antibodies; Sebia HYDRASYS protein electrophoresis system was used for oligoclonal band electrophoresis; BD FACSCanto II flow cytometer and its reagents were used to analyze lymphocyte subpopulations; KingMed and Newterrain were used to test AQP4-IgG.

### 2.3 Statistical analysis

SPSS (version 26.0) and GraphPad Prism (version 9.0) were used for statistical analysis. Categorical data were expressed as numbers and percentages, and comparisons were assessed using the chi-square test. For normally distributed continuous data, results are presented as mean and standard deviations (SD), while non-normally distributed data are presented as median and range. Comparisons of continuous variables were evaluated using the t-test. Significance was defined as a P-value of <0.05 (two-tailed).

### 2.4 Establishment and validation of the model

To ensure a transparent and reproducible variable-selection strategy for model development, a multi-step screening procedure was applied. First, clinical characteristics, imaging features, and laboratory variables showing significant differences between AQP4-IgG-negative NMOSD and other CNS autoimmune diseases (P < 0.05) were identified through univariate group comparisons. These candidate variables were then individually evaluated using univariate logistic regression, and variables with P < 0.05 were entered into a multivariable logistic regression model. Finally, variables that remained independently associated with AQP4-IgG-negative NMOSD (P < 0.05) in the multivariable analysis were retained for model construction.

False discovery rate (FDR) correction (Benjamini–Hochberg method) was applied only to descriptive univariate comparisons to control for multiple testing. FDR-adjusted significance was not used as the sole criterion for model inclusion, because statistical significance after correction does not necessarily indicate independent predictive value in multivariable settings.

Missing data were handled using multiple imputation by chained equations (MICE), under the assumption that data were missing at random. Multiple imputed datasets were generated, and the completed data were subsequently used for model development and validation. To assess model robustness and reduce overfitting, a 10-fold cross-validation strategy was applied within the training cohort.

The entire dataset was randomly divided into a training set and a validation set at a ratio of 7:3, with stratification to preserve outcome proportions. Model training and feature selection were performed exclusively in the training set, whereas the validation set was reserved for independent performance evaluation.

Five machine-learning algorithms were implemented to construct predictive models, including logistic regression, random forest, support vector machine, k-nearest neighbors, and gradient boosting methods. Model performance was evaluated using the area under the receiver operating characteristic curve and additional diagnostic metrics. All statistical analyses and model development procedures were conducted using R software (version 4.5.1).

## 3 Result

### 3.1 Comparison of demographic characteristics

The AQP4-IgG-negative NMOSD group had a male-to-female ratio of 0.60, comprising 32 males and 53 females. The age of onset ranged from 3 to 68 years, with a mean of 39.59 ± 15.16 years. The AQP4-IgG-positive NMOSD group comprised 24 males and 168 females, with a male-to-female ratio of 0.14. The age of onset in this group also ranged from 3 to 68 years, with a median age of onset was 44.5 (31.25, 56) years. The other nervous system autoimmune disorders control group had a male-to-female ratio of 1.10, comprising 287 males and 260 females. In this group, the age of onset ranged from 4 to 85 years, with a median age of 39.00 (27.00, 54.00) years. The healthy control group had a male-to-female ratio of 0.40, with 77 males and 192 females and a median age of 40 (33, 48.5) years.

In the AQP4-IgG-negative NMOSD group, the male-to-female ratio was significantly higher than that in the AQP4-IgG-positive NMOSD group (P < 0.0001) but lower than that in the disease control group (P = 0.0140). The age at onset in the AQP4-IgG-negative NMOSD group was significantly lower than that in the AQP4-IgG-positive NMOSD group (P = 0.0440), whereas no significant difference was observed compared with the disease control group (P = 0.8432) (**Table 1**).

**Table 1:** Comparison of demographic characteristics among the four groups.

Characteristics	The AQP 4-IgG-negative NMOSD group	The AQP 4-IgG-positive NMOSD group (1)	Other nervous system autoimmune disorders Control group (2)	Health control group (3)	*P*1	*P*2	*P*3
Sex					< 0.0001 *	0.0140 *	0.1380
Male	32 (37.65)	24 (12.50)	287 (52.47)	77 (28.62)			
Female	53 (62.35)	168 (87.50)	260 (47.53)	192 (71.38)			
Years	38 (25, 58)	44.5 (31.25, 56)	39 (27, 54)	40 (33, 48.5)	0.0440 *	0.8432	0.5950
Height	1.650 (1.6, 1.7)	1.600 (1.580, 1.665)	1.670 (1.600, 1.730)	–	0.0038 *	0.0313 *	–
Weight	64 (55.00, 74.75)	60 (52.38, 70.00)	65 (58.00, 75.00)	–	0.1142	0.2904	–
BMI	23.50 (20.17, 26.34)	23.01 (20.61, 26.04)	23.44 (20.96, 26.12)	23.40 (21.60, 25.20)	0.5417	0.9811	0.9544

*is P <0.05; P1 indicates the P value obtained between the AQP 4-IgG-negative NMOSD group with the AQP 4-IgG-positive NMOSD group; P2 indicates the P value obtained between the AQP 4-IgG-negative NMOSD group and other CNS control groups; and P3 represents the P value obtained between the AQP 4-IgG-negative NMOSD group and the healthy control group.

### 3.2 Comparison of clinical characteristics

Ocular symptoms were observed in 49 cases (57.65%), limb sensory abnormalities in 65 cases (76.47%), limb weakness in 61 cases (71.76%), urinary and fecal dysfunction in 31 cases (36.47%), gastrointestinal symptoms in 33 cases (38.82%), dizziness in 25 cases (29.41%), diplopia in 30 cases (35.29%), swallowing and choking difficulties in 17 cases (20%), facial sensory disturbances in 23 cases (27.06%), ear symptoms in 5 cases (5.88%), somnolence in 3 cases (3.53%), fever in 11 cases (12.94%), headache in 18 cases (21.18%), language disorders in 18 cases (21.18%), decreased level of consciousness in 3 cases (3.53%), and seizures in 8 cases (9.41%) in the AQP4-IgG-negative NMOSD group. The incidence rates of limb sensory abnormalities (89.58%) and urinary and fecal dysfunction (57.29%) were significantly higher in the AQP4-IgG-positive NMOSD group than in the AQP4-IgG-negative NMOSD group, with statistically significant differences between the two groups. Conversely, the incidence rates of diplopia (17.19%), facial sensory disturbances (15.10%), headache (10.94%), and language disorders (7.81%) were significantly lower in the AQP4-IgG-positive NMOSD group than in the AQP4-IgG-negative NMOSD group, also showing statistically significant differences. Compared with the other nervous system autoimmune disorders control groups, the incidence of decreased level of consciousness (17.37%) was significantly higher in the AQP4-IgG-negative NMOSD group, with statistically significant differences observed. Additionally, the incidence rates of ocular symptoms (18.28%), urinary and fecal dysfunction (21.21%), gastrointestinal symptoms (15.72%), diplopia (17.18%), and swallowing and choking difficulties (10.42%) were significantly lower in the AQP4-IgG-negative NMOSD group than in the nervous system autoimmune disorders control groups (**Table 2**).

**Table 2:** Comparison of clinical characteristics between the three disease groups.

Characteristics	The AQP 4 -IgG-negative NMOSD group	The AQP 4-IgG-positive NMOSD group (1)	other nervous system autoimmune disorders Control group (2)	*P*1	*P*2
Ocular symptoms				0.7940	<0.0001 *
Yes	49 (57.65)	107 (55.73)	100 (18.28)		
No	36 (42.35)	85 (44.27)	447 (81.72)		
Limb sensory abnormalities				0.0056 *	0.1028
Yes	65 (76.47)	172 (89.58)	368 (67.28)		
No	20 (23.53)	20 (10.42)	179 (32.72)		
Limb weakness				0.2161	0.3232
Yes	61 (71.76)	152 (79.17)	360 (65.81)		
No	24 (28.24)	40 (20.83)	187 (34.19)		
Urinary and fecal dysfunction				0.0017 *	0.0035 *
Yes	31 (36.47)	110 (57.29)	116 (21.21)		
No	54 (63.53)	82 (42.71)	431 (78.79)		
Gastrointestinal symptoms				0.4986	<0.0001 *
Yes	33 (38.82)	66 (34.38)	86 (15.72)		
No	52 (61.18)	126 (65.63)	461 (84.28)		
Dizziness				0.1268	0.1249
Yes	25 (29.41)	40 (20.83)	118 (21.57)		
No	60 (70.59)	152 (79.17)	429 (78.43)		
Diplopia				0.0017 *	0.0003 *
Yes	30 (35.29)	33 (17.19)	94 (17.18)		
No	55 (64.71)	159 (82.81)	453 (82.82)		
Water swallowing and choking difficulties				0.4916	0.0171 *
Yes	17 (20.00)	31 (16.15)	57 (10.42)		
No	68 (80.00)	161 (83.85)	490 (89.58)		
Facial sensory disturbances				0.0292 *	0.4963
Yes	23 (27.06)	29 (15.10)	129 (23.58)		
No	62 (72.94)	163 (84.90)	418 (76.42)		
Ear symptoms				0.1386	0.3634
Yes	5 (5.88)	4 (2.08)	20 (3.66)		
No	80 (94.12)	188 (97.92)	527 (96.34)		
Somnolence				>0.9999	0.7424
Yes	3 (3.53)	7 (3.65)	17 (3.11)		
No	82 (96.47)	185 (96.35)	530 (96.89)		
Fever				0.5297	0.6286
Yes	11 (12.94)	19 (9.90)	87 (15.90)		
No	74 (87.06)	173 (90.10)	460 (84.10)		
Headache				0.0379 *	0.3536
Yes	18 (21.18)	21 (10.94)	91 (16.64)		
No	67 (78.82)	171 (89.06)	456 (83.36)		
Language disorders				0.0024 *	0.2738
Yes	18 (21.18)	15 (7.81)	88 (16.09)		
No	67 (78.82)	177 (92.19)	459 (83.91)		
Decreased level of consciousness				0.5653	0.0003 *
Yes	3 (3.53)	12 (6.25)	95 (17.37)		
No	82 (96.47)	180 (93.75)	452 (82.63)		
Seizures				0.3042	>0.9999
Yes	8 (9.41)	11 (5.73)	50 (9.14)		
No	77 (90.59)	181 (94.27)	497 (90.86)		

*is P <0.05; P1 indicates the P value obtained comparing the AQP 4-IgG-negative NMOSD group with the AQP 4-IgG-positive NMOSD group; P2 indicates the P value obtained comparing the AQP 4-IgG-negative NMOSD group with other CNS control groups.

### 3.3 Comparison of magnetic resonance imaging characteristics

In the AQP4-IgG-negative NMOSD group, involvement was predominantly observed in the brainstem (50.59%, 43/85) and spinal cord (70.59%, 60/85), with varying degrees of involvement in the cerebral lobes (29.42%, 25/85), centrum semiovale (9.41%, 8/85), cerebellum (11.76%, 10/85), thalamus (18.82%, 16/85), and basal ganglia (17.65%, 15/85). The AQP4-IgG-positive NMOSD group showed a significantly higher prevalence of lesions in the spinal cord (88.02%, 169/192) than the AQP4-IgG-negative NMOSD group, a statistically significant difference. Conversely, prevalence rates were significantly lower in the brainstem (31.77%, 61/192), cerebellum (4.69%, 9/192), thalamus (6.25%, 12/192), and basal ganglia (5.21%, 10/192). Compared with the other nervous system autoimmune disorders control groups, the cerebral lobes (54.48%, 298/547) and centrum semiovale (20.29%, 111/547) showed significantly higher prevalence rates, whereas the brainstem (28.70%, 157/547), thalamus (7.5%, 41/547), and spinal cord (38.57%, 211/547) showed significantly lower rates. No statistically significant intergroup differences in cumulative imaging locations were observed when compared with the AQP4-IgG-negative NMOSD group (P_1_ = 0.0062, P_2_ = 0.2966). In summary, compared to the AQP4-IgG-negative NMOSD group, the AQP4-IgG-positive NMOSD group showed significantly increased imaging accumulation sites (P = 0.0062) (**Table 3**).

**Table 3:** Comparison of MR imaging characteristics among the three affected groups.

Characteristics	The AQP 4-IgG-negative NMOSD group	The AQP 4-IgG-positive NMOSD group (1)	Other nervous system autoimmune disorders Control group (2)	*P*1	*P*2
Cumulative site	2. (1, 3)	2 (1, 3)	2 (1, 4)	0.0062*	0.2966
Cerebral lobes				0.5609	<0.0001*
Yes	25 (29.41)	50 (26.04)	298 (54.48)		
No	60 (70.59)	142 (73.96)	249 (45.52)		
Ventricles				0.1589	0.0904
Yes	24 (28.24)	38 (19.79)	209 (38.21)		
No	61 (71.76)	154 (80.21)	338 (61.79)		
Centrum semiovale				>0.9999	0.0165*
Yes	8 (9.41)	20 (10.42)	111 (20.29)		
No	77 (90.59)	172 (89.58)	436 (79.71)		
Corona radiata				0.2046	0.7502
Yes	12 (14.12)	17 (8.85)	88 (16.09)		
No	73 (85.88)	175 (91.15)	459 (83.91)		
Capsula interna				0.4634	0.1057
Yes	4 (4.71)	5 (2.60)	10 (1.83)		
No	81 (95.29)	187 (97.40)	537 (98.17)		
Brainstem				0.0045*	<0.0001*
Yes	43 (50.59)	61 (31.77)	157 (28.70)		
No	42 (49.41)	131 (68.23)	390 (71.30)		
Epencephala				0.0400*	0.7025
Yes	10 (11.76)	9 (4.69)	56 (10.24)		
No	75 (88.24)	183 (95.31)	491 (89.76)		
Cerebral ganglia				0.0023*	0.0018*
Yes	16 (18.82)	12 (6.25)	41 (7.5)		
No	69 (81.18)	180 (93.75)	506 (92.50)		
Basal ganglia				0.0023*	0.4165
Yes	15 (17.65)	10 (5.21)	79 (14.44)		
No	70 (82.35)	182 (94.79)	468 (85.56)		
Callosum				0.0753	0.8557
Yes	10 (11.76)	10 (5.21)	62 (11.33)		
No	75 (88.24)	182 (94.79)	485 (88.67)		
Spinal cord				0.0009*	<0.0001*
Yes	60 (70.59)	169 (88.02)	211 (38.57)		
No	25 (29.41)	23 (11.98)	336 (61.43)		

*is P <0.05; P1 indicates the P value obtained comparing the AQP 4 -IgG-negative NMOSD group with the AQP 4-IgG-positive NMOSD group; P2 indicates the P value obtained comparing the AQP 4-IgG-negative NMOSD group with other CNS control groups.

### 3.4 Comparison of serum lipid indices

The differences in levels of triglycerides, total cholesterol, high-density lipoprotein (HDL), low-density lipoprotein (LDL), apolipoprotein A1, and apolipoprotein B between the AQP4-IgG-positive NMOSD and AQP4-IgG-negative NMOSD groups were not statistically significant (P > 0.05). Compared with the AQP4-IgG-negative NMOSD group, the nervous system autoimmune disorders control group showed no statistically significant differences in triglyceride, LDL, and apolipoprotein B levels (P = 0.9428, P = 0.2176, and P = 0.7085, respectively); however, total cholesterol, HDL, and apolipoprotein A1 levels were significantly lower (P = 0.0379, P = 0.0115, and P = 0.0117, respectively). The healthy control group showed significantly higher HDL and apolipoprotein A1 levels than the AQP4-IgG-negative NMOSD group and significantly lower levels of triglycerides, apolipoprotein B, and LDL. All five indicators were statistically significant, as detailed in the attached table (**Supplementary Table S1**).

### 3.5 Comparison of autoimmune antibody indicators

The positivity rates of SS-A and RO-52 antibodies were significantly higher in the AQP4-IgG-positive NMOSD group than in the AQP4-IgG-negative NMOSD group, with statistically significant differences (P = 0.0005 and P = 0.0003, respectively); however, differences in other antibody levels were not statistically significant (P > 0.05), as detailed in the table. The positivity rate for mitochondrial M2 subtype antibodies was significantly higher in the other nervous system autoimmune disorders control group than in the AQP4-IgG-negative NMOSD group (P = 0.0406). Differences in other antibody levels were not statistically significant (P > 0.05), as detailed in the attached table (**Supplementary Table S2**).

### 3.6 Comparison of hematological and inflammatory indicators

The absolute values of eosinophils, absolute values of basophils, relative values of eosinophils, absolute values of red blood cells, and hemoglobin levels in the AQP4-IgG-positive NMOSD group were significantly lower than those in the AQP4-IgG-negative NMOSD group (P = 0.0092, P = 0.0021, P = 0.0392, P = 0.0422, and P = 0.0131, respectively); other hematological parameters were not significantly different.

Compared to the AQP4-IgG-negative NMOSD group, the other nervous system autoimmune disorders control group had significantly higher absolute values of eosinophils, absolute values of basophils, relative values of eosinophils, and relative values of basophils, with statistically significant differences (P = 0.0307, P = 0.0489, P = 0.0122, and P = 0.0006). Absolute values of neutrophils, relative values of neutrophils, and red blood cell distribution width were significantly lower, with statistically significant differences (P = 0.0219, P = 0.0154, and P = 0.0031, respectively).

In the comparison between the AQP4-IgG-positive NMOSD group and AQP4-IgG-negative NMOSD group, no statistically significant differences were observed in the systemic immune inflammation index (SII), neutrophil-lymphocyte ratio (NLR), platelet-to-lymphocyte ratio (PLR), lymphocyte-to-monocyte ratio (LMR), or neutrophil-to-platelet ratio (NPR). However, significant differences in NLR and NPR levels (P = 0.0111 and P = 0.0012, respectively) were observed between the other nervous system autoimmune disorders control group and the AQP4-IgG-negative NMOSD group, whereas SII, PLR, and LMR levels were not statistically significant (P > 0.05) (**Table 4**).

**Table 4:** Comparison of blood routine and inflammatory indexes among the four groups.

Characteristics	The AQP 4-IgG-negative NMOSD group	The AQP 4-IgG-positive NMOSD group (1)	Other nervous system autoimmune disorders Control group (2)	Health Control group (3)	*P*1	*P*2	*P*3
White blood cell	7.130 (5.265, 9.680)	6.615 (5.245, 8.828)	6.510 (5.290, 8.300)	5.470 (4.785, 6.370)	0.2320	0.0544	< 0.0001*
Leukomonocyte	1.770 (1.390, 2.345)	1.745 (1.263, 2.153)	1.860 (1.460, 2.350)	1.750 (1.510, 2.048)	0.2492	0.6178	0.5013
Monocyte	0.4300 (0.3300, 0.5650)	0.3900 (0.2900, 0.5275)	0.4100 (0.3200, 0.5400)	0.2900 (0.2500, 0.3625)	0.0911	0.4212	< 0.0001*
Neutrophile	4.580 (3.050, 6.565)	4.225 (3.180, 6.230)	3.860 (2.950, 5.380)	3.270 (2.713, 3.975)	0.6191	0.0219*	< 0.0001*
Eosinocyte	0.0700 (0.0300, 0.1600)	0.0500 (0.0100, 0.0900)	0.1000 (0.0500, 0.1700)	0.1000 (0.0600, 0.1500)	0.0092*	0.0307*	< 0.0001*
Basicyte	0.0200 (0.0100, 0.0300)	0.0100 (0.0100, 0.0200)	0.0200 (0.0100, 0.0300)	0.0200 (0.0200, 0.0400)	0.0021	0.0489*	< 0.0001*
SII	631.6 (332.9, 893.2)	525.9 (312.7, 985.4)	475.2 (327.4, 746.8)	477.3 (343.9, 614.9)	0.6060	0.1442	0.0054*
NLR	2.490 (1.745, 3.850)	2.520 (1.680, 3.918)	2.050 (1.470, 3.000)	1.910 (1.480, 2.363)	0.8580	0.0111*	< 0.0001*
PLR	123.5 (94.66, 172.1)	127.6 (89.81, 179.2)	127.7 (99.36, 166.4)	143.1 (115.4, 169.6)	0.6077	0.4032	0.0058*
LMR	4.190 (3.030, 5.395)	4.225 (3.208, 6.030)	4.570 (3.370, 5.940)	6.015 (4.830, 7.368)	0.4227	0.1126	< 0.0001*
NPR	0.02 (0.01, 0.03)	0.02 (0.01, 0.03)	0.02 (0.01, 0.02)	0.01 (0.01, 0.02)	0.1697	0.0012*	< 0.0001*

*is P <0.05; P1 indicates the P value obtained comparing the AQP 4-IgG-negative NMOSD group with the AQP 4-IgG--positive NMOSD group; P2 indicates the P value obtained comparing the AQP 4-IgG-negative NMOSD group with other CNS control groups; P2 indicates the P value obtained comparing the AQP 4-IgG-negative NMOSD group with health Control group.

### 3.7 Comparison of the oligoclonal zone electrophoresis and 24-hour intrathecal protein synthesis rate

The results for cerebrospinal fluid (CSF) IgG oligoclonal bands (P_1_ = 0.7704, P = 0.2793), serum IgG oligoclonal bands (P_1_ = 0.8932, P = 0.9840), and CSF-specific IgG oligoclonal bands (P_1_ = 0.9239, P = 0.1017) showed no statistically significant differences among the AQP4-IgG-negative NMOSD group, AQP4-IgG-positive NMOSD group, and other nervous system autoimmune disorders control groups.

The AQP4-IgG-positive NMOSD group showed significantly elevated levels of CSF and serum IgG compared to the AQP4-IgG-negative NMOSD group (P = 0.0294, P = 0.0262). However, no statistically significant differences were observed in the intrathecal IgG synthesis rate, CSF albumin, or serum albumin levels (P = 0.7189, P = 0.3195, P = 0.0504).

The nervous system autoimmune disorders control group showed significantly higher rates of intrathecal IgG synthesis and CSF albumin, CSF IgG, and serum IgG levels (P < 0.0001, P = 0.0027, P < 0.0001, and P = 0.0003, respectively) than the AQP4-IgG-negative NMOSD group, with no statistically significant difference in serum albumin levels (P = 0.2757) (**Table 5**).

**Table 5:** Comparison of OCB and 24h ISR.

Characteristics	The AQP 4-IgG-negative NMOSD group	The AQP 4-IgG-positive NMOSD group (1)	other nervous system autoimmune disorders Control group (2)	*P*1	*P*2
CSF IgG oligoclonal band				0.7704	0.2793
+	35 (41.18)	63 (37.06)	259 (50.29)		
±	11 (12.94)	21 (12.35)	51 (9.90)		
–	39 (45.88)	86 (50.59)	205 (39.81)		
Serum IgG oligoclonal band				0.8932	0.9840
+	12 (14.12)	21 (12.35)	71 (13.79)		
±	7 (8.24)	16 (9.41)	40 (7.77)		
–	66 (77.65)	133 (78.24)	404 (78.45)		
CSF-specific IgG oligoclonal band				0.9239	0.1017
+	27 (31.76)	52 (30.59)	218 (42.33)		
±	9 (10.59)	16 (9.41)	32 (6.21)		
–	49 (58.33)	102 (61.45)	265 (51.46)		
Intrathecal IgG synthesis rate	2.515 (0.6450, 4.735)	2.320 (0.4000, 5.810)	6.790 (1.960, 14.68)	0.7189	< 0.0001*
CSF albumin	0.2150 (0.1300, 0.3150)	0.2200 (0.1700, 0.3000)	0.2500 (0.1700, 0.4500)	0.3195	0.0027*
Serum albumin	40.15 (36.38, 43.10)	38.40 (35.40, 42.10)	40.90 (36.60, 44.00)	0.0504	0.2757
CSF IgG	0.0285 (0.0215, 0.0470)	0.0340 (0.0240, 0.0550)	0.0480 (0.0310, 0.0870)	0.0294*	< 0.0001*
Serum IgG	9.580 (8.398, 12.38)	10.70 (9.000, 13.80)	11.00 (9.500, 13.50)	0.0262*	0.0003*

*is P <0.05; P1 indicates the P value obtained comparing the AQP 4-IgG-negative NMOSD group with the AQP 4-IgG-positive NMOSD group; P2 indicates the P value obtained comparing the AQP 4-IgG-negative NMOSD group with other CNS control groups.

### 3.8 FDR correction for different indicators

In the unadjusted analyses, several variables including Neutrophile count, total bilirubin, Eosinocyte count, Height, total cholesterol (TC), anti-mitochondrial M2 isoform antibody, albumin/globulin ratio, and Basicyte count showed statistically significant differences between groups (all P < 0.05). However, after applying FDR correction at the 0.1 threshold, these associations did not remain significant (all FDR-adjusted P ≥ 0.1, all q ≥ 0.1: e.g., neutrophil count q=0.128, total cholesterol q=0.115).Variables remaining significant after FDR correction included: ocular symptoms (q=0.0119), NLR (q=0.069521), HDL cholesterol (q=0.068425), sex (q=0.075727), Anti mitochondrial M2 isoform antibody (q=0.161047), apolipoprotein A1 (q=0.0663), neutrophile (q=0.104244),total bilirubin (q=0.106642), eosinocyte (q=0.135307), height (q=0.133025), total cholesterol (TC, q=155521) and albumin/globulin ratio (q=0.160074) (**Supplementary Table S3**). After applying the false discovery rate (FDR) correction (q < 0.1), variables that remained statistically significant included diplopia, brainstem involvement, spinal cord involvement, serum albumin, and CSF albumin, indicating their robustness as distinguishing features.

### 3.9 Establishment and validation of differential diagnostic model

**Figure 1:**
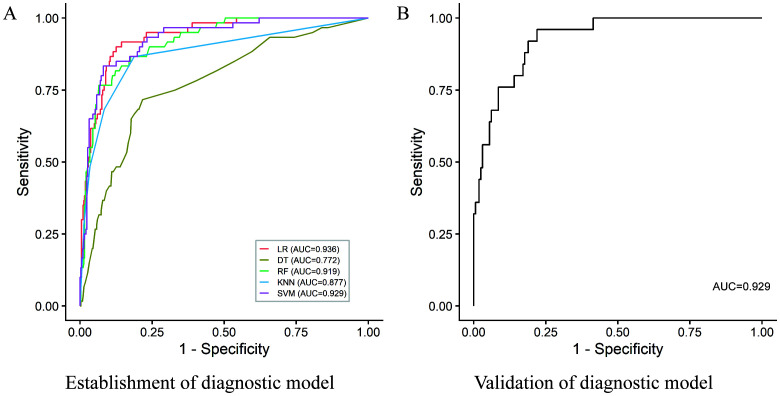
Receiver operating characteristic (ROC) curves of the diagnostic models for differentiating AQP4-IgG–negative NMOSD from other nervous system autoimmune disorders. **(A)** ROC curves of different machine-learning models in the training cohort, including logistic regression (LR), random forest (RF), support vector machine (SVM), k-nearest neighbors (KNN), and decision tree (DT). **(B)** ROC curve of the final logistic regression model in the validation cohort.

As is shown in **Figure 1** and **Supplementary Tables S4-6**, twelve indicators (Ocular symptoms, Gastrointestinal symptoms, Water swallowing and choking difficulties, Cerebral lobes, Centrum semiovale, Brainstem, Cerebral ganglia, Spinal cord, APOA1, Ca, Toxoplasma gondii IgM Antibody, CSF IgG) were finally selected. Five machine learning algorithms, including logistic regression, decision tree, random forest, k-nearest neighbors, and support vector machine, were systematically compared under the same cross-validation framework. Among them, the Logistic Regression model demonstrated the best overall performance, with an AUC of 0.936, accuracy of 0.878 and specificity of 0.875 in the internal training cohort, indicating excellent ability to identify negative samples while maintaining acceptable sensitivity (recall=0.900). Validation confirmed the robustness and generalizability of the model, with an AUC of 0.929 accuracy of 0.868, specificity of 0.884, and recall of 0.603.

Importantly, compared with more complex machine-learning approaches, the LR model provided superior interpretability and clinical applicability, allowing explicit estimation of disease probability and definition of a clinically meaningful decision threshold. This balance between diagnostic performance and transparency supports its suitability as a practical tool for clinical decision-making.

Given the heterogeneity of the comparator group, additional disease-specific analyses were performed to evaluate the discriminative ability of the final logistic regression model. Separate binary classifications were conducted between AQP4-IgG-negative NMOSD and each individual diagnostic category, including multiple sclerosis, Guillain–Barré syndrome, viral encephalitis, and autoimmune encephalitis. The model consistently demonstrated high diagnostic performance across these pairwise comparisons, with AUC values exceeding 0.94 for all disease categories (details shown in **Supplementary Figure S1** and **Supplementary Table S7**). The explicit logistic regression equation, including coefficient estimates, probability calculation, and the recommended decision cutoff, is provided in the Supplementary Appendix.

### 3.10 Trend test for AQP4-IgG-negative NMOSD group

**Figure 2:**
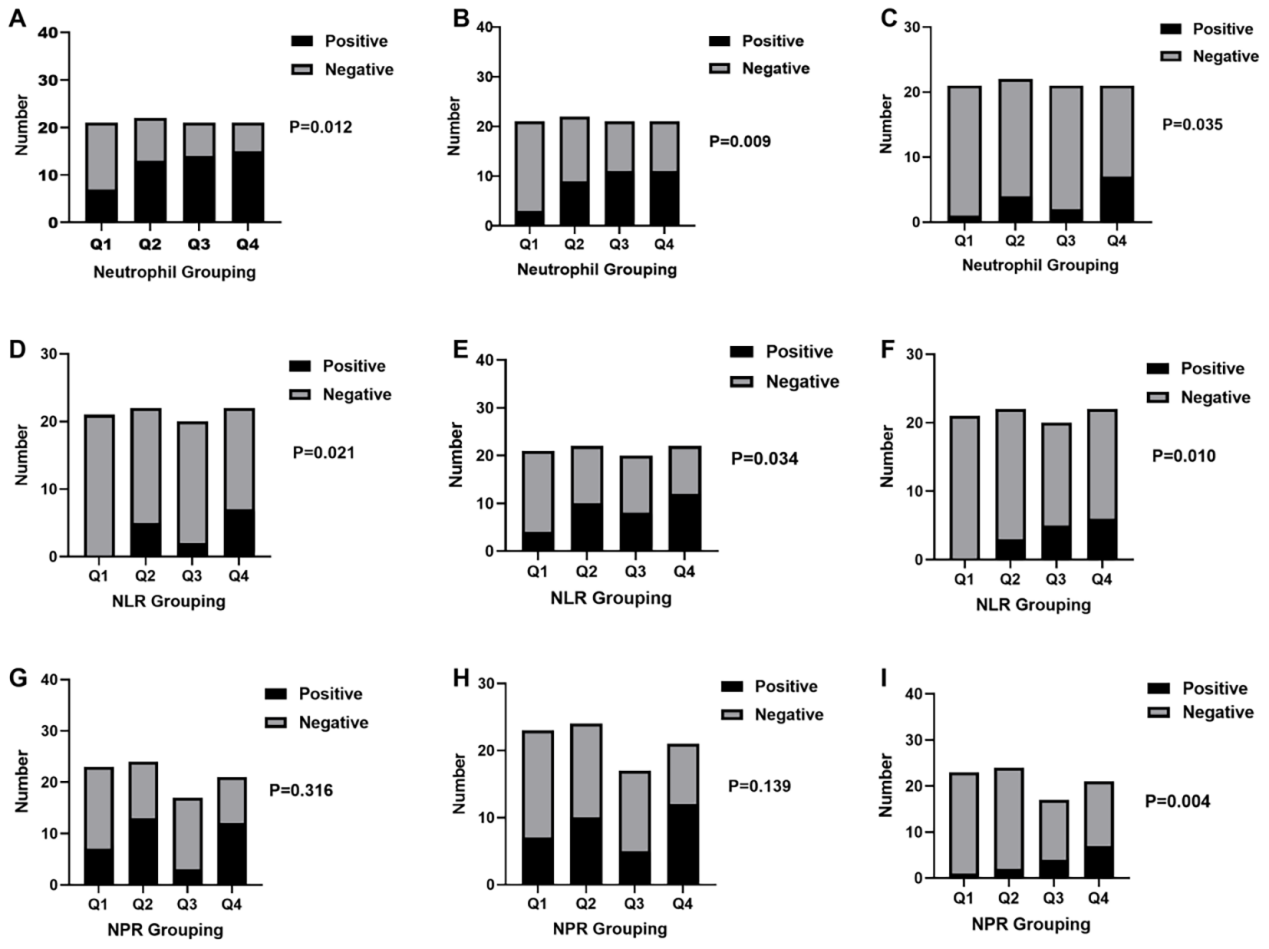
Trend analysis of inflammatory indices in patients with AQP4-IgG–negative NMOSD. Patients with AQP4-IgG–negative NMOSD were stratified into quartiles (Q1–Q4) according to neutrophil count **(A–C)**, neutrophil-to-lymphocyte ratio [NLR; **(D–F)**], and neutrophil-to-platelet ratio [NPR; **(G–I)**]. For each quartile, stacked bar charts display the number of patients with (“Positive”, black bars) and without (“Negative”, gray bars) the corresponding clinical or imaging features. Specifically: **(A, D, G)** optic neuritis; **(B, E, H)** presence of ≥4 clinical manifestations; **(C, F, I)** presence of ≥5 cumulative MRI lesion sites. P values were calculated using the Cochran–Armitage trend test to assess whether increasing levels of inflammatory indices were associated with a higher probability of the corresponding outcomes.

**Figure 2** and **Supplementary Table S8** show a significant correlation between optic neuritis and neutrophil count (P = 0.012) when patients are grouped by neutrophil count. As neutrophil count increases, the likelihood of optic neuritis also increases. A significant correlation was also observed between acute disseminated encephalomyelitis and NLR (P = 0.021). As NLR increases, the probability of acute disseminated encephalomyelitis also increases.

## 4 Discussion

NMOSD is an autoimmune inflammatory demyelinating disease of the central nervous system, most commonly affecting the optic nerves and spinal cord. While AQP4-IgG is a highly specific diagnostic biomarker, a subset of patients remains seronegative, posing substantial diagnostic challenges in clinical practice (13, 18, 19). In this study, we systematically compared clinical features, imaging findings, and laboratory parameters of AQP4-IgG–negative NMOSD with other nervous system autoimmune disorders to identify distinguishing indicators.

Several autoimmune and inflammatory CNS disorders share overlapping clinical manifestations with NMOSD, which contributes to diagnostic challenges in AQP4-IgG–negative patients. This underscores the need for integrative diagnostic approaches based on routinely available clinical and laboratory indicators, as explored in the present study (14).

Our results demonstrate that AQP4-IgG–negative NMOSD exhibits a distinct clinical and radiological profile. Compared with AQP4-IgG–positive NMOSD, seronegative patients showed a lower frequency of spinal cord involvement and related symptoms, such as limb sensory disturbance and urinary dysfunction, but a higher prevalence of brainstem involvement and associated manifestations including diplopia. These findings suggest a different lesion distribution pattern, which may partially explain the observed clinical heterogeneity (20).

Imaging analysis further supported these differences, with AQP4-IgG–negative NMOSD showing predominant brainstem involvement and moderate spinal cord lesions compared with AQP4-IgG–positive cases (21, 22). In contrast, other nervous system autoimmune disorders were more frequently characterized by supratentorial brain involvement. These imaging features provide practical clues for differential diagnosis, particularly in patients with negative AQP4-IgG testing.

Laboratory analyses revealed additional distinguishing features. Alterations in inflammatory indices, including neutrophil-related parameters, were observed in AQP4-IgG–negative NMOSD, reflecting an active inflammatory state (5, 23). Trend analyses further indicated associations between inflammatory markers and clinical severity, supporting their potential role as auxiliary indicators. However, these markers should be interpreted as reflecting overall inflammatory burden rather than disease-specific pathology (24).

Studies have shown that lipoproteins participate in lipid metabolism and play roles in inflammatory responses in the body (25). This study identified significant differences in various lipid parameters, including triglycerides, HDL, LDL, apolipoprotein A1, and apolipoprotein B. HDL, known for its anti-atherosclerotic effects, has been extensively studied for its pivotal role in immune modulation and inflammation suppression. Altered HDL and apolipoprotein A1 levels may reflect differences in inflammatory regulation between AQP4-IgG–negative NMOSD and control groups (26).

Compared to prior biomarkers: (1) GFAP (a marker of astrocyte injury) was not analyzed in our study, but our data show milder CSF IgG elevation vs. AQP4-IgG-positive NMOSD. Because CSF IgG elevation can arise from blood-CSF barrier dysfunction, intrathecal synthesis, or generalized inflammatory activity, this finding likely reflects a relatively lower degree of CNS inflammatory response rather than direct astrocyte injury; GFAP, a well-recognized marker of astrocyte damage, was not assessed in our cohort. Therefore, while the observed differences in CSF IgG levels indicate varying inflammatory burden and barrier integrity, they should not be interpreted as a quantitative measure of astrocytic destruction. (2) IL-6 was not measured, but elevated NLR implies Th17 activation (a key source of IL-6), consistent with IL-6’s role in NMOSD pathogenesis; (3) Complement C3/C4 levels were not assessed, but lower SS-A antibody positivity in AQP4-IgG-negative NMOSD suggests less overlap with systemic autoimmune disorders, unlike complement activation in AQP4-IgG-positive cases.

Although NMOSD and multiple sclerosis are both central nervous system demyelinating disorders, they are characterized by distinct immunopathological profiles, which may contribute to differences in inflammatory activity and associated laboratory findings (26).

Although the prevalence of risk factors increases with age, the lipid profile differences between the two are not directly age-related. Instead, they are driven by each’s unique immunoregulatory abnormalities—such as interferon-β affecting cholesterol levels in MS and anti-aquaporin-4 antibody-mediated lesions impacting lipid metabolism in NMOSD—both independent of age (27).

Epidemiological data have shown that even after adjusting for age, the lipid parameters of the two diseases still maintain significant differences (28). Specifically, interferon-β, a commonly used drug in MS treatment, can reduce cholesterol synthesis by regulating hepatic enzyme activity, thereby affecting serum cholesterol levels; in NMOSD, anti-aquaporin-4 antibody-mediated lesions can disrupt the blood-brain barrier, leading to the leakage of plasma lipids into the cerebrospinal fluid, and also affect the expression of lipid transporters in neural cells, resulting in abnormal lipid accumulation in the central nervous system (29). Both mechanisms are independent of age, further confirming that the lipid metabolism differences between the two diseases are rooted in their distinct pathological processes.

In clinical practice, distinguishing AQP4-IgG–negative NMOSD from other autoimmune and infectious CNS diseases relies on integrating serological and imaging findings (30, 31). Our study highlights that clinical, imaging, and laboratory indicators can effectively support differential diagnosis without overreliance on disease-specific rare biomarkers.

Importantly, we developed and validated a multi-parameter diagnostic model incorporating routinely available laboratory indicators (32). The model demonstrated good discriminative performance and high specificity, supporting its potential utility as an adjunctive tool in the evaluation of patients with suspected AQP4-IgG–negative NMOSD. Such an approach may help reduce misdiagnosis in clinically ambiguous cases (33).

This study has limitations, including its retrospective design, single-center cohort, and incomplete testing for MOG-IgG in all seronegative patients. Future multicenter prospective studies incorporating additional disease-specific biomarkers are needed to further refine diagnostic strategies.

In conclusion, AQP4-IgG–negative NMOSD displays distinct clinical, imaging, and laboratory characteristics compared with other nervous system autoimmune disorders. A combined diagnostic approach integrating multiple routinely available indicators may enhance diagnostic accuracy in seronegative patients.

## Declarations

All serum samples were obtained from Beijing Tiantan Hospital, Capital Medical University, China. This study utilized residual samples collected after patients’ formal examinations at the hospital, ensuring that no additional procedures were performed on the patients. Patient confidentiality was strictly maintained, and no personal information was disclosed in this article. The study was approved by the Medical Ethics Committee of Beijing Tiantan Hospital, Capital Medical University (Approval No. KY2023-157-03).

## Data Availability

The raw data supporting the conclusions of this article will be made available by the authors, without undue reservation.
