# Supplementary material for: Diagnostic biomarkers for differentiating AQP4-IgG- negative NMOSD from other nervous system autoimmune disorders: a retrospective study
Source: Front Immunol. 2026 Jan 14;16:1637613. doi: 10.3389/fimmu.2025.1637613 (PMC12846999; doi:10.3389/fimmu.2025.1637613)
Supplement: Supplementary file 1 [file Table1.docx]

**Diagnostic Biomarkers for Differentiating AQP4-IgG-Negative NMOSD from Other CNS Disordersnervous system autoimmune disorders: A Retrospective Study**

2.2 Research Methodology

2.2.1 Clinical information collection

General information was collected for four groups, including gender, age, marital status, education level, birthplace, blood pressure, BMI, heart rate, and presence of underlying conditions (such as hypertension, diabetes, cardiovascular disease, and cerebrovascular disease). The initial symptoms, clinical manifestations, and magnetic resonance imaging (MRI) data (including brain MRI and spinal cord MRI) of patients from the AQP4 IgG-negative NMOSD group, AQP4 IgG-positive NMOSD group, and the other CNS disease control group were recorded and analyzed.

2.2.2 Clinical and Imaging Indicators Classification

Clinical manifestations were categorized according to the NMOSD diagnostic criteria established by the international diagnostic panel into 6 groups of core clinical syndromes (optic neuritis, acute myelitis, area postrema syndrome, acute brainstem syndrome, acute diencephalic syndrome, and cerebral syndrome).

In order to enhance the precision and granularity of the analysis, the initial categorization of six clinical symptoms was refined into sixteen more specific and targeted clinical manifestations, corresponding to each of the aforementioned core categories: ocular symptoms (corresponding to optic neuritis), abnormal limb sensation, limb weakness, urinary and fecal incontinence (corresponding to acute myelitis), gastrointestinal symptoms (corresponding to area postrema syndrome), dizziness, diplopia, dysphagia and choking on liquids, facial sensory disturbances, ear symptoms (corresponding to acute brainstem syndrome), hypersomnia, fever (corresponding to acute diencephalic syndrome), headache, language impairment, decreased consciousness level, seizures (corresponding to cerebral syndrome).

For other central nervous system diseases, their clinical features are not entirely identical to NMOSD, but their clinical manifestations can be categorized into the aforementioned 16 clinical manifestations. Therefore, the same classification framework was employed during the analysis and discussion to ensure the systematic and consistent nature of the study. In the analysis of magnetic resonance imaging (MRI) data, after referring to relevant literature, Based on brain anatomical structures, the regions affected by MRI lesions corresponding to the six core clinical symptoms were classified into 10 regions (lobes, ventricles, centrum semiovale, corona radiata, internal capsule, brainstem, cerebellum, thalamus, basal ganglia, and corpus callosum). Special attention was also given to key regions of the cervical and thoracic spinal cord in the spinal cord MRI analysis.

2.2.3 Instruments and reagents

Using the Mindray BC-6900 fully automated hematology analyzer and its reagents, blood routine related indicators (Neu, Lym, plt, SD) were tested; the Mindray EH2080C fully automated urine analyzer was used to test urine routine related indicators (urine specific gravity, urine pH); indirect immunofluorescence assays employing Euroimmun reagents from Germany were used to detect autoantibodies; Beckman DXI800 fully automated chemiluminescence analyzer was used to measure thyroid function indicators (T3, T4, FT3, FT4, TSH); Hitachi LABOSPECT008 fully automated biochemical analyzer was used to measure biochemical parameters (CK, IBIL, DBIL, UA, Alb); Roche e602 fully automated immunoassay analyzer was used to detect EB virus antibodies and herpes simplex virus antibodies; Sebia HYDRASYS protein electrophoresis system was used for oligoclonal band electrophoresis; BD FACSCanto II flow cytometer and its reagents were used to analyze lymphocyte subpopulations; KingMed and Newterrain were uesd to test AQP4 IgG.

Peripheral blood routine parameters (including Neu, Lym, Plt, and SD) were measured using the Mindray BC-6900 fully automated hematology analyzer, and urine routine indices (urine specific gravity and pH) were assessed with the Mindray EH2080C automated urine analyzer. Thyroid function markers (T3, T4, FT3, FT4, and TSH) were quantified with the Beckman DXI800 chemiluminescence immunoassay analyzer. Biochemical parameters (CK, IBIL, DBIL, UA, and Alb) were determined by the Hitachi LABOSPECT008 automated biochemical analyzer. Antibodies against Epstein–Barr virus and herpes simplex virus were tested with the Roche e602 immunoassay analyzer. Autoantibodies were screened by indirect immunofluorescence assays using Euroimmun reagents (Germany). Lymphocyte subpopulations were analyzed by flow cytometry with a BD FACSCanto II instrument and corresponding reagents. Oligoclonal bands in cerebrospinal fluid (CSF) were examined using the Sebia HYDRASYS protein electrophoresis system. Serum AQP4-IgG antibodies were tested by certified external laboratories (KingMed and Newterrain) using standardized cell-based assays (CBA). All assays were performed in accordance with the manufacturers’ protocols, and cut-off values recommended by the kits were applied to define seropositivity.

Brain and spinal cord MRI scans were reviewed independently by two experienced neuroradiologists who were blinded to antibody status. Lesions were assessed in terms of anatomical location, distribution, and morphology according to the international NMOSD imaging criteria.

Cerebrospinal fluid (CSF) samples were collected by lumbar puncture under sterile conditions. Routine parameters including cell count, protein concentration, and glucose levels were analyzed immediately after collection or storage at 4℃ within one day. Oligoclonal bands were further assessed by isoelectric focusing with immunoblotting. All CSF samples requiring additional testing were processed following standardized laboratory protocols.

2.3 Statistical analysis

SPSS (version 26.0) and GraphPad Prism (version 9.0) were used for statistical analysis. Categorical data were expressed as numbers and percentages, and comparisons were assessed using the chi-square test. For normally distributed continuous data, results are presented as mean and standard deviations (SD), while non-normally distributed data are presented as median and range. Comparisons of continuous variables were evaluated using the t-test. Significance was defined as a P-value of <0.05 (two-tailed).


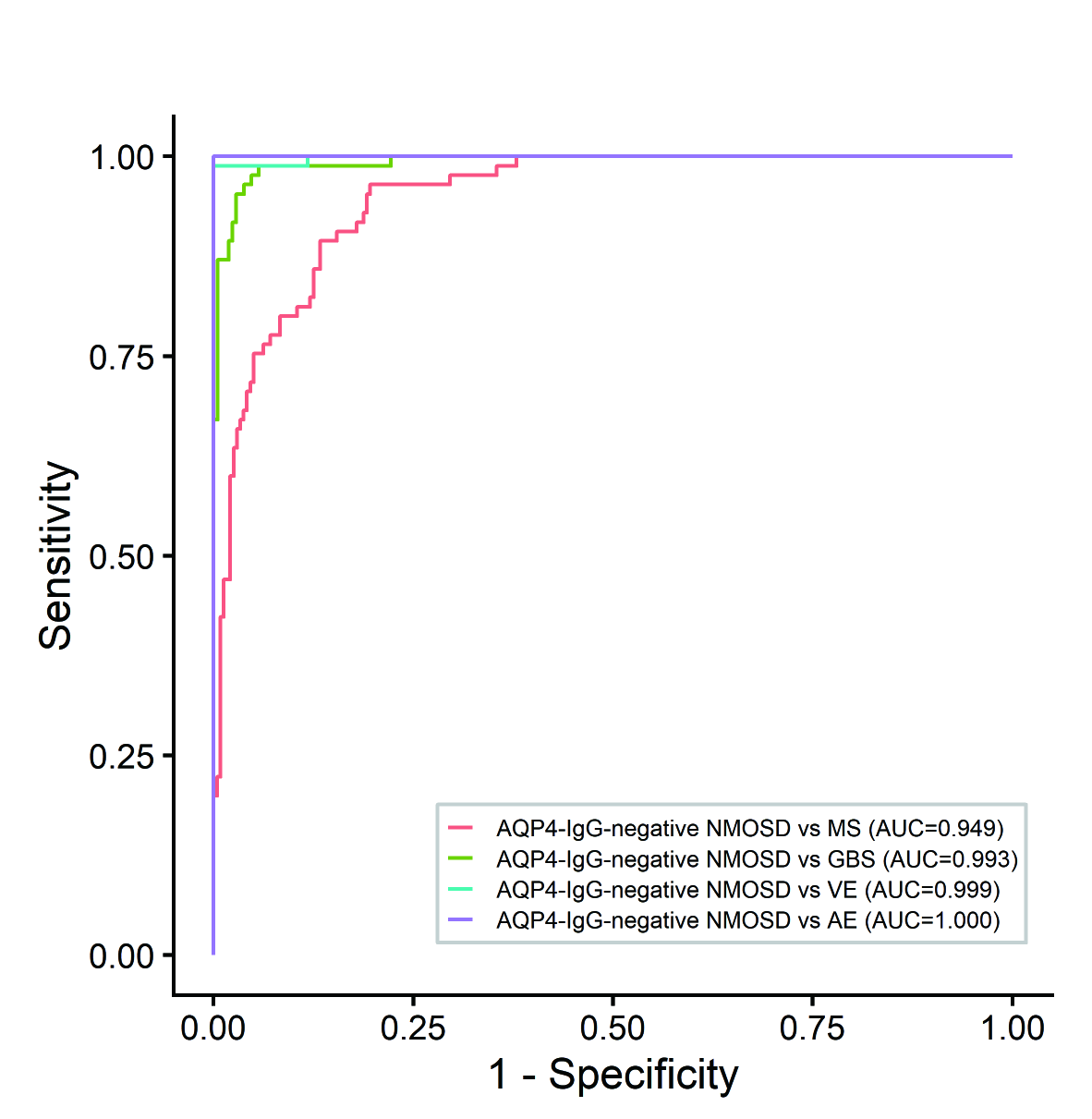


Figure S1 Disease-specific analyses of the proposed model

TableS1 Comparison of serum biochemical indicators indexes among the four groups

| Characteristics | The AQP 4 antibody-negative NMOSD group | The AQP 4 antibody-positive NMOSD group（1） | Other nervous system autoimmune disorders  control group（2） | Health  control group（3） | *P*1 | *P*2 | *P*3 |
| --- | --- | --- | --- | --- | --- | --- | --- |
| Dyslipidemia index | 2.094±1.493 | 2（1，3） | 2（1，3） | 0（0，0） | 0.5344 | 0.3061 | 0.1586 |
| Triglyceride(TG) | 1.210（0.7650，1.830） | 1.260（0.8400，1.790） | 1.180（0.8400，1.758） | 0.9300（0.6800，1.210） | 0.8599 | 0.9428 | < 0.0001* |
| Total cholesterol(TC) | 4.491±1.021 | 4.550（3.770，5.220） | 4.175（3.620，4.818） | 4.453±0.5427 | 0.5536 | 0.0379* | 0.3956 |
| HDL cholesterol | 1.331±0.3834 | 1.290（1.050，1.593） | 1.170（0.9800，1.410） | 1.562±0.2782 | 0.8107 | 0.0115 | < 0.0001* |
| LDL cholesterol | 2.660（2.070，3.255） | 2.715（2.143，3.415） | 2.490（2.070，3.090） | 2.562±0.4848 | 0.5264 | 0.2176 | 0.0252* |
| APOA1 | 1.290（1.083，1.533） | 1.290（1.100，1.540） | 1.220（1.050，1.410） | 1.530±0.2117 | 0.9706 | 0.0117* | < 0.0001* |
| AOPB | 0.8630±0.2477 | 0.8300（0.7100，1.020） | 0.8200（0.6900，1.000） | 0.7300（0.6600，0.8100） | 0.6764 | 0.7085 | < 0.0001* |
| ALT | 17.15（11.83，31.98） | 16.80（12.00，25.20） | 19.10（12.50，36.90） | 15.30（11.60，22.05） | 0.4831 | 0.3436 | 0.0379* |
| AST | 16.10（12.88，20.63） | 16.00（13.60，19.90） | 17.40（14.10，23.15） | 17.20（14.90，19.90） | 0.8769 | 0.0853 | 0.1853 |
| LDH | 175.5（156.8，208.1） | 175.2（152.9，208.3） | 170.0（149.7，198.7） | 163.0（150.9，180.1） | 0.6274 | 0.1259 | 0.0003* |
| ALP | 59.20（45.70，72.43） | 59.50（49.90，73.75） | 62.40（49.80，77.45） | 56.90（48.40，68.30） | 0.5520 | 0.1399 | 0.6698 |
| GGT | 22.30（13.55，40.95） | 20.05（14.38，32.95） | 21.00（13.40，36.45） | 15.30（11.90，21.65） | 0.5811 | 0.7689 | < 0.0001* |
| Total protein | 64.50（61.00，67.90） | 64.90（61.53，69.60） | 66.50（63.35，71.55） | 71.80±3.118 | 0.3545 | 0.0003* | < 0.0001* |
| Albumin | 40.15±3.061 | 39.63±3.874 | 40.80（38.20，42.90） | 44.66±1.989 | 0.2767 | 0.2590 | < 0.0001* |
| Globulin | 23.95（21.83，27.70） | 25.20（22.33，28.35） | 25.00（23.00，28.75） | 27.14±2.389 | 0.1075 | 0.0045* | < 0.0001* |
| Albumin/globulin ratio | 1.667±0.2951 | 1.600（1.400，1.800） | 1.600（1.400，1.800） | 1.600（1.500，1.800） | 0.0578 | 0.0417* | 0.2892 |
| Total bilirubin | 9.350（7.070，12.33） | 9.000（7.000，11.50） | 10.45（8.100，13.63） | 11.50（9.350，14.18） | 0.4739 | 0.0233* | < 0.0001* |
| K | 3.845（3.610，4.083） | 3.950（3.710，4.243） | 3.940（3.740，4.160） | 4.172±0.2752 | 0.0253* | 0.0513 | < 0.0001 |
| Na | 140.0（138.2，143.0） | 141.0（138.6，142.0） | 140.8（138.9，142.0） | 140.4±1.932 | 0.9038 | 0.8848 | 0.4964 |
| CL | 103.2（101.0，106.0） | 104.0（102.0，106.5） | 104.0（101.0，106.0） | 104.6±1.951 | 0.0481* | 0.4537 | 0.0005* |
| Ca | 2.306±0.09969 | 2.321±0.1033 | 2.340（2.270，2.410） | 2.403±0.08079 | 0.2707 | 0.0041* | < 0.0001* |
| P | 1.260（1.140，1.398） | 1.240（1.130，1.370） | 1.250（1.130，1.400） | 1.080（0.9900，1.180) | 0.4142 | 0.8187 | < 0.0001* |

Note: * is P <0.05; P1 indicates the P value obtained between the AQP 4-antibody-negative NMOSD group with the AQP 4-positive NMOSD group; P2 indicates the P value obtained between the AQP 4-antibody-negative NMOSD group and other CNS control groups; and P3 represents the P value obtained between the AQP 4-antibody-negative NMOSD group and the healthy control group

TableS2 Comparison of autoimmune antibody indicators among the three affected groups

| Characteristics | The AQP 4 antibody-negative NMOSD group | The AQP 4 antibody-positive NMOSD group (1) | Other nervous system autoimmune disorders  Control group (2) | *P*1 | *P*2 |
| --- | --- | --- | --- | --- | --- |
| Anti neutrophil cytoplasmic antibody profile |  |  |  | 1.0000 | 0.0704 |
| + | 3（4.00） | 7（3.83） | 5（0.98） |  |  |
| - | 72（96.00） | 176（96.17） | 505（99.02） |  |  |
| Neutrophil cytoplasmic antibodies (Perinuclear type) |  |  |  | 1.0000 | 0.2227 |
| + | 2（2.67） | 5（2.73） | 5（0.98） |  |  |
| - | 73（97.33） | 178（97.27） | 505（99.02） |  |  |
| Neutrophil cytoplasmic antibodies (Cytoplasmic type) |  |  |  | 1.0000 | 0.1282 |
| + | 1（1.33） | 2（1.09） | 0（0.00） |  |  |
| - | 74（98.67） | 181（98.91） | 510（100.00） |  |  |
| Anti nRNP antibody |  |  |  | 0.1067 | >0.9999 |
| + | 2（2.44） | 15（7.81） | 12（2.37） |  |  |
| - | 80（97.56） | 177（92.19） | 495（97.63） |  |  |
| Anti SM antibody |  |  |  | 1.0000 | 0.3627 |
| + | 1（1.22） | 4（2.17） | 2（0.39） |  |  |
| - | 81（98.78） | 184（97.83） | 505（99.61） |  |  |
| Anti SS-A antibody |  |  |  | 0.0005* | 0.4766 |
| + | 8（9.76） | 54（29.35） | 66（13.02） |  |  |
| - | 74（90.24） | 130（70.65） | 441（86.98） |  |  |
| Anti SS-B antibody |  |  |  | 0.1823 | >0.9999 |
| + | 1（1.22） | 9（4.89） | 6（1.18） |  |  |
| - | 81（98.78） | 175（95.11） | 501（98.82） |  |  |
| Anti SCL-70 antibody |  |  |  | 1.0000 | 0.5949 |
| + | 1（1.22） | 3（1.63） | 5（0.99） |  |  |
| - | 81（98.78） | 181（98.37） | 502（99.01） |  |  |
| Anti PM-SCL antibody |  |  |  | 1.0000 | 0.4519 |
| + | 1（1.22） | 3（1.63） | 3（0.59） |  |  |
| - | 81（98.78） | 181（98.37） | 504（99.41） |  |  |
| Anti JO-1 antibody |  |  |  | 1.0000 | 0.0952 |
| + | 2（2.44） | 4（2.17） | 2（0.39） |  |  |
| - | 80（97.56） | 180（97.83） | 505（99.61） |  |  |
| Anticentromere antibody |  |  |  | 0.1117 | 0.0578 |
| + | 0（0.0） | 8（4.35） | 22（4.34） |  |  |
| - | 82（100.0） | 176（95.65） | 485（95.66） |  |  |
| Antibody to proliferating cell nuclear antigen |  |  |  | 0.5893 | 0.1979 |
| + | 2（2.44） | 2（1.09） | 4（0.79） |  |  |
| - | 80（97.56） | 182（98.91） | 503（99.21） |  |  |
| Anti dsDNA antibody |  |  |  | 1.0000 | >0.9999 |
| + | 0（0.0） | 1（0.54） | 3（0.59） |  |  |
| - | 82（100.0） | 183（99.46） | 504（99.41） |  |  |
| Histonic antibody |  |  |  | 0.2824 | >0.9999 |
| + | 1（1.22） | 8（4.35） | 10（1.97） |  |  |
| - | 81（98.78） | 176（95.65） | 497（98.03） |  |  |
| Anti ribosomal P protein antibody |  |  |  | 1.0000 | 0.5949 |
| + | 1（1.22） | 3（1.63） | 5（0.99） |  |  |
| - | 81（98.78） | 181（98.37） | 502（99.01） |  |  |
| Anti mitochondrial M2 isoform antibody |  |  |  | 0.1126 | 0.0406* |
| + | 4（4.88） | 21（11.41） | 65（12.82） |  |  |
| - | 78（95.12） | 163（88.59） | 442（87.18） |  |  |
| Antimyocardial antibody |  |  |  | 1.0000 | >0.9999 |
| + | 0（0.0） | 1（0.55） | 2（0.39） |  |  |
| - | 82（100.0） | 182（99.45） | 505（99.61） |  |  |
| Anti gastric parietal cells  Antibody |  |  |  | 0.1816 | 0.6012 |
| + | 0（0.0） | 6（3.28） | 7（1.38） |  |  |
| - | 82（100.0） | 177（96.72） | 500（98.62） |  |  |
| Smooth muscle antibody |  |  |  | 1.0000 | >0.9999 |
| + | 0（0.0） | 1（0.55） | 3（0.59） |  |  |
| - | 82（100.0） | 182（99.45） | 504（99.41） |  |  |
| Anti mitochondrial antibody |  |  |  | 0.3145 | >0.9999 |
| + | 0（0.0） | 4（2.19） | 6（1.18） |  |  |
| - | 82（100.0） | 179（97.81） | 501（98.82） |  |  |
| Anti liver and renal microsomal antibodies |  |  |  | 1.0000 | >0.9999 |
| + | 0（0.0） | 0（0.0） | 0（0.00） |  |  |
| - | 82（100.0） | 183（100.0） | 507（100.00） |  |  |
| RO-52 |  |  |  | 0.0003* | 0.3022 |
| + | 8（10.13） | 56（30.43） | 76（15.23） |  |  |
| - | 71（89.87） | 128（69.57） | 423（84.77） |  |  |
| Nucleosome antibody |  |  |  | 0.5113 | 0.2553 |
| + | 1（1.27） | 1（0.54） | 1（0.2） |  |  |
| - | 78（98.73） | 183（99.46） | 497（99.80） |  |  |
| Herpes Simplex Virus Type I IgG Antibody |  |  |  | 1.0000 | 0.0521 |
| + | 25（51.02） | 51（50.50） | 62（26.16） |  |  |
| - | 24（48.98） | 50（49.50） | 175（73.84） |  |  |
|  |  |  |  |  |  |
| Rubella Virus IgG Antibody |  |  |  | 0.2969 | 0.0501 |
| + | 23（46.94） | 58（56.86） | 70（29.05） |  |  |
| - | 26（53.06） | 44（43.14） | 171（70.95） |  |  |
|  |  |  |  |  |  |
| Rubella Virus IgM Antibody |  |  |  | 1.0000 | >0.9999 |
| + | 0（0.00） | 1（0.99） | 1（0.42） |  |  |
| - | 49（100.00） | 100（99.01） | 239（99.58） |  |  |
|  |  |  |  |  |  |
| Toxoplasma gondii IgG Antibody |  |  |  | 0.1876 | 0.2401 |
| + | 3（4.17） | 14（9.72） | 20（3.41） |  |  |
| - | 69（95.83） | 130（90.28） | 396（96.59） |  |  |
|  |  |  |  |  |  |
| EB Virus Capsid Antigen IgG Antibody |  |  |  | 0.1138 | >0.9999 |
| + | 35（47.95） | 87（59.59） | 175（41.57） |  |  |
| - | 38（52.05） | 59（40.41） | 246（58.43） |  |  |
|  |  |  |  |  |  |
| EB Virus Capsid Antigen IgM Antibody |  |  |  | 0.3034 | >0.9999 |
| + | 0（0.00） | 4（2.76） | 5（1.18） |  |  |
| - | 73（100.00） | 141（97.24） | 418（98.82） |  |  |
|  |  |  |  |  |  |
| EB Virus Early Antigen IgM Antibody |  |  |  | 1.0000 | 0.1143 |
| + | 0（0.00） | 1（0.68） | 183（19.48） |  |  |
| - | 72（100.00） | 146（99.32） | 243（80.52） |  |  |
|  |  |  |  |  |  |
| EB Virus Nuclear Antigen IgG Antibody |  |  |  | 0.1965 | 0.4936 |
| + | 33（45.83） | 81（55.48） | 171（39.52） |  |  |
| - | 39（54.17） | 65（44.52） | 254（60.48） |  |  |
|  |  |  |  |  |  |
| Toxoplasma gondii IgM Antibody |  |  |  | 1.0000 | <0.0001 |
| + | 0（0.00） | 2（1.36） | 153（37.59） |  |  |
| - | 73（100.00） | 145（98.64） | 254（62.41） |  |  |
|  |  |  |  |  |  |
| Cytomegalovirus IgG Antibody |  |  |  | 0.1553 | 0.1436 |
| + | 55（79.71） | 125（87.41） | 155（37.26） |  |  |
| - | 14（20.29） | 18（12.59） | 261（62.74） |  |  |
|  |  |  |  |  |  |
| Cytomegalovirus IgM Antibody |  |  |  | 0.5525 | 0.3664 |
| + | 0（0.00） | 3（2.05） | 9（2.27） |  |  |
| - | 73（100.00） | 143（97.95） | 387（97.73） |  |  |
|  |  |  |  |  |  |
| Herpes Simplex Virus Type II IgG Antibody |  |  |  | 0.7513 | 0.4062 |
| + | 3（6.12） | 9（9.09） | 12（3.35） |  |  |
| - | 46（93.88） | 90（90.91） | 346（96.65） |  |  |
|  |  |  |  |  |  |
| Coxsackie Virus IgM Antibody |  |  |  | 1.0000 | >0.9999 |
| + | 0（0.00） | 0（0.0） | 0（0.00） |  |  |
| - | 73（100.00） | 147（100.0） | 421（100.00） |  |  |
| Cerebrospinal Fluid IgG Oligoclonal Bands |  |  |  | 0.7704 | 0.2793 |
| + | 35 | 63 | 259 |  |  |
| ± | 11 | 21 | 51 |  |  |
| - | 39 | 86（50.59） | 205 |  |  |
|  |  |  |  |  |  |
| Serum IgG Oligoclonal Bands |  |  |  | 0.8932 | 0.9840 |
| + | 12 | 21 | 71 |  |  |
| ± | 7 | 16 | 40 |  |  |
| - | 66 | 133 | 404 |  |  |
|  |  |  |  |  |  |
| Cerebrospinal Fluid-specific IgG Oligoclonal Bands |  |  |  | 0.9239 | 0.1017 |
| + | 27 | 52 | 218 |  |  |
| ± | 9 | 16 | 32 |  |  |
| - | 49 | 102（61.45） | 265 |  |  |
| Alpha-fetoprotein（AFP） | 2.890（2.190，4.000） | 2.770（1.960，3.765） | 2.660（1.870，3.515） | 0.5156 | 0.0720 |
| Carcinoembryonic antigen（CEA） | 2.075±1.130 | 1.610（0.9785，2.403） | 1.665（1.048，2.563） | 0.1460 | 0.2953 |
| Total cerebrospinal fluid cell count | 11.00（4.000，108.5） | 14.00（6.000，109.0） | 11.00（3.000，112.0） | 0.3188 | 0.7771 |
| Cerebrospinal fluid white blood cell count | 6.000（3.000，11.00） | 7.000（3.500，13.00） | 4.000（2.000，11.00） | 0.2752 | 0.1749 |
| Vitamin B12 | 847.0（561.3，1308） | 844.0（544.0，1710） | 693.0（428.5，1124） | 0.4533 | 0.1004 |
| corticosteroid treatment |  |  |  | 0.1027 | 0.002* |
| + | 73 (85.88) | 177 (92.19) | 361 (66.00) |  |  |
| - | 12 (4.12) | 15 (7.81) | 186 (34.00) |  |  |

Note: * is P <0.05; P1 indicates the P value obtained comparing the AQP 4 antibody-negative NMOSD group with the AQP 4 antibody-positive NMOSD group; P2 indicates the P value obtained comparing the AQP 4 antibody-negative NMOSD group with other CNS control groups

Table S3 FDR correction of differential diagnostic indicators

|  | p | priority | q（adjusted p-value） |
| --- | --- | --- | --- |
| Ocular symptoms | 0.0001 | 1 | 0.0119 |
| Gastrointestinal symptoms | 0.0001 | 2 | 0.00595 |
| Cerebral lobes | 0.0001 | 3 | 0.003967 |
| Brainstem | 0.0001 | 4 | 0.002975 |
| Spinal cord | 0.0001 | 5 | 0.00238 |
| Intrathecal IgG synthesis rate | 0.0001 | 6 | 0.001983 |
| CSF IgG | 0.0001 | 7 | 0.0017 |
| Toxoplasma gondii IgM Antibody | 0.0001 | 8 | 0.001488 |
| Diplopia | 0.0003 | 9 | 0.003967 |
| Decreased level of consciousness | 0.0003 | 10 | 0.00357 |
| Serum IgG | 0.0003 | 11 | 0.003245 |
| Total protein | 0.0003 | 12 | 0.002975 |
| NPR | 0.0012 | 13 | 0.010985 |
| Cerebral ganglia | 0.0018 | 14 | 0.0153 |
| CSF albumin | 0.0027 | 15 | 0.02142 |
| Urinary and fecal dysfunction | 0.0035 | 16 | 0.026031 |
| Ca | 0.0041 | 17 | 0.0287 |
| GLB | 0.0045 | 18 | 0.02975 |
| NLR | 0.0111 | 19 | 0.069521 |
| HDL cholesterol | 0.0115 | 20 | 0.068425 |
| APOA1 | 0.0117 | 21 | 0.0663 |
| Sex | 0.014 | 22 | 0.075727 |
| Centrum semiovale | 0.0165 | 23 | 0.08537 |
| Water swallowing and choking difficulties | 0.0171 | 24 | 0.084788 |
| Neutrophile | 0.0219 | 25 | 0.104244 |
| Total bilirubin | 0.0233 | 26 | 0.106642 |
| Eosinocyte | 0.0307 | 27 | 0.135307 |
| Height | 0.0313 | 28 | 0.133025 |
| Total cholesterol(TC) | 0.0379 | 29 | 0.155521 |
| Anti mitochondrial M2 isoform antibody | 0.0406 | 30 | 0.161047 |
| Albumin/globulin ratio | 0.0417 | 31 | 0.160074 |
| Basicyte | 0.0489 | 32 | 0.181847 |
| Rubella Virus IgG Antibody | 0.0501 | 33 | 0.180664 |
| K | 0.0513 | 34 | 0.17955 |
| Herpes Simplex Virus Type I IgG Antibody | 0.0521 | 35 | 0.17714 |
| White blood cell | 0.0544 | 36 | 0.179822 |
| Anticentromere antibody | 0.0578 | 37 | 0.185897 |
| Anti neutrophil cytoplasmic antibody profile | 0.0704 | 38 | 0.220463 |
| Alpha-fetoprotein（AFP） | 0.072 | 39 | 0.219692 |
| AST | 0.0853 | 40 | 0.253768 |
| Ventricles | 0.0904 | 41 | 0.26238 |
| Anti JO-1 antibody | 0.0952 | 42 | 0.269733 |
| Vitamin B12 | 0.1004 | 43 | 0.277851 |
| CSF-specific IgG oligoclonal band | 0.1017 | 44 | 0.275052 |
| Cerebrospinal Fluid-specific IgG Oligoclonal Bands | 0.1017 | 45 | 0.26894 |
| Limb sensory abnormalities | 0.1028 | 46 | 0.265939 |
| Capsula interna | 0.1057 | 47 | 0.267623 |
| LMR | 0.1126 | 48 | 0.279154 |
| EB Virus Early Antigen IgM Antibody | 0.1143 | 49 | 0.277586 |
| Dizziness | 0.1249 | 50 | 0.297262 |
| LDH | 0.1259 | 51 | 0.293767 |
| Neutrophil cytoplasmic antibodies (Cytoplasmic type) | 0.1282 | 52 | 0.293381 |
| ALP | 0.1399 | 53 | 0.314115 |
| Cytomegalovirus IgG Antibody | 0.1436 | 54 | 0.316452 |
| SII | 0.1442 | 55 | 0.311996 |
| Cerebrospinal fluid white blood cell count | 0.1749 | 56 | 0.371663 |
| Antibody to proliferating cell nuclear antigen | 0.1979 | 57 | 0.41316 |
| LDL cholesterol | 0.2176 | 58 | 0.446455 |
| Neutrophil cytoplasmic antibodies (Perinuclear type) | 0.2227 | 59 | 0.449175 |
| Toxoplasma gondii IgG Antibody | 0.2401 | 60 | 0.476198 |
| Nucleosome antibody | 0.2553 | 61 | 0.498044 |
| Albumin | 0.259 | 62 | 0.497113 |
| Language disorders | 0.2738 | 63 | 0.517178 |
| Serum albumin | 0.2757 | 64 | 0.51263 |
| CSF IgG oligoclonal band | 0.2793 | 65 | 0.511334 |
| Cerebrospinal Fluid IgG Oligoclonal Bands | 0.2793 | 66 | 0.503586 |
| Weight | 0.2904 | 67 | 0.515785 |
| Carcinoembryonic antigen（CEA） | 0.2953 | 68 | 0.516775 |
| Cumulative site | 0.2966 | 69 | 0.511528 |
| RO-52 | 0.3022 | 70 | 0.51374 |
| Dyslipidemia index | 0.3061 | 71 | 0.513041 |
| Limb weakness | 0.3232 | 72 | 0.534178 |
| ALT | 0.3436 | 73 | 0.560115 |
| Headache | 0.3536 | 74 | 0.568627 |
| Anti SM antibody | 0.3627 | 75 | 0.575484 |
| Ear symptoms | 0.3634 | 76 | 0.569008 |
| Cytomegalovirus IgM Antibody | 0.3664 | 77 | 0.566255 |
| PLR | 0.4032 | 78 | 0.615138 |
| Herpes Simplex Virus Type II IgG Antibody | 0.4062 | 79 | 0.611871 |
| Basal ganglia | 0.4165 | 80 | 0.619544 |
| Monocyte | 0.4212 | 81 | 0.6188 |
| Anti PM-SCL antibody | 0.4519 | 82 | 0.655806 |
| CL | 0.4537 | 83 | 0.650486 |
| Anti SS-A antibody | 0.4766 | 84 | 0.675183 |
| EB Virus Nuclear Antigen IgG Antibody | 0.4936 | 85 | 0.69104 |
| Facial sensory disturbances | 0.4963 | 86 | 0.686741 |
| Anti SCL-70 antibody | 0.5949 | 87 | 0.813714 |
| Anti ribosomal P protein antibody | 0.5949 | 88 | 0.804467 |
| Anti gastric parietal cells | 0.6012 | 89 | 0.803852 |
| Leukomonocyte | 0.6178 | 90 | 0.816869 |
| Fever | 0.6286 | 91 | 0.822015 |
| Epencephala | 0.7025 | 92 | 0.908668 |
| AOPB | 0.7085 | 93 | 0.906575 |
| Somnolence | 0.7424 | 94 | 0.939847 |
| Corona radiata | 0.7502 | 95 | 0.939724 |
| GGT | 0.7689 | 96 | 0.953116 |
| Total cerebrospinal fluid cell count | 0.7771 | 97 | 0.953349 |
| P | 0.8187 | 98 | 0.994136 |
| Year | 0.8432 | 99 | 1.013543 |
| Callosum | 0.8557 | 100 | 1.018283 |
| Na | 0.8848 | 101 | 1.042487 |
| Triglyceride(TG) | 0.9428 | 102 | 1.099933 |
| BMI | 0.9811 | 103 | 1.133504 |
| Serum IgG oligoclonal band | 0.984 | 104 | 1.125923 |
| Serum IgG Oligoclonal Bands | 0.984 | 105 | 1.1152 |
| Seizures | 0.9999 | 106 | 1.122529 |
| Anti nRNP antibody | 0.9999 | 107 | 1.112038 |
| Anti SS-B antibody | 0.9999 | 108 | 1.101742 |
| Anti dsDNA antibody | 0.9999 | 109 | 1.091634 |
| Histonic antibody | 0.9999 | 110 | 1.08171 |
| Antimyocardial antibody | 0.9999 | 111 | 1.071965 |
| Smooth muscle antibody | 0.9999 | 112 | 1.062394 |
| Anti mitochondrial antibody | 0.9999 | 113 | 1.052992 |
| Anti liver and renal microsomal antibodies | 0.9999 | 114 | 1.043755 |
| Rubella Virus IgM Antibody | 0.9999 | 115 | 1.034679 |
| EB Virus Capsid Antigen IgG Antibody | 0.9999 | 116 | 1.025759 |
| EB Virus Capsid Antigen IgM Antibody | 0.9999 | 117 | 1.016992 |
| Coxsackie Virus IgM Antibody | 0.9999 | 118 | 1.008374 |
| Antibody |  | 119 | 0 |

Note: Values with p < 0.0001 are sorted by 0.0001, and those with p > 0.9999 are sorted by 0.9999. Correction was performed at the FDR level of 0.1.

Table S4 Univariate logistic regression

| Variables | beta | S.E | Z | OR[95%CI] | P_value |
| --- | --- | --- | --- | --- | --- |
| SEX (Overall) |  |  |  |  | 0.011 |
| Height | -2.454 | 1.179 | -2.082 | 0.086 (0.009–0.866) | 0.037 |
| Ocular.symptoms | 1.632 | 0.243 | 6.707 | 5.113 (3.174–8.237) | 0.000 |
| Urinary.and.fecal.dysfunction | 0.758 | 0.248 | 3.049 | 2.133 (1.311–3.471) | 0.002 |
| Gastrointestinal.symptoms | 1.224 | 0.252 | 4.865 | 3.402 (2.077–5.571) | 0.000 |
| Diplopia (Overall) |  |  |  |  | 0.000 |
| Water.swallowing.and.choking.difficulties | 0.765 | 0.305 | 2.507 | 2.149 (1.182–3.908) | 0.012 |
| Decreased.level.of.consciousness | -1.748 | 0.599 | -2.921 | 0.174 (0.054–0.563) | 0.003 |
| Cerebral.lobes | -1.243 | 0.260 | -4.788 | 0.288 (0.173–0.480) | 0.000 |
| Centrum.semiovale | -1.070 | 0.408 | -2.619 | 0.343 (0.154–0.764) | 0.009 |
| Brainstem (Overall) |  |  |  |  | 0.000 |
| Cerebral.ganglia | 0.980 | 0.325 | 3.019 | 2.665 (1.410–5.038) | 0.003 |
| Spinal.cord | 1.393 | 0.269 | 5.187 | 4.027 (2.379–6.818) | 0.000 |
| Total.protein | -0.065 | 0.019 | -3.429 | 0.937 (0.902–0.972) | 0.001 |
| Globulin | -0.072 | 0.024 | -2.997 | 0.931 (0.888–0.975) | 0.003 |
| Total.bilirubin | -0.063 | 0.027 | -2.326 | 0.939 (0.891–0.990) | 0.020 |
| HDL.cholesterol | 1.086 | 0.344 | 3.161 | 2.962 (1.511–5.809) | 0.002 |
| APOA1 | 1.285 | 0.406 | 3.167 | 3.613 (1.631–8.001) | 0.002 |
| Ca | -2.384 | 0.953 | -2.501 | 0.092 (0.014–0.597) | 0.012 |
| White.blood.cell | 0.084 | 0.038 | 2.211 | 1.087 (1.010–1.171) | 0.027 |
| Neutrophile | 0.094 | 0.039 | 2.390 | 1.098 (1.017–1.186) | 0.017 |
| NPR | 22.793 | 6.970 | 3.270 | 7920077498.079 (9247.558–6783155844285597.000) | 0.001 |
| Anti.mitochondrial.M2.isoform.antibody | -0.607 | 0.308 | -1.973 | 0.545 (0.298–0.996) | 0.048 |
| Toxoplasma.gondii.IgM.Antibody | -2.144 | 0.413 | -5.197 | 0.117 (0.052–0.263) | 0.000 |
| Intrathecal.IgG.synthesis.rate | -0.016 | 0.006 | -2.632 | 0.984 (0.972–0.996) | 0.008 |
| CSF.albumin | -2.194 | 0.663 | -3.308 | 0.111 (0.030–0.409) | 0.001 |
| CSF.IgG | -26.388 | 5.278 | -4.999 | 0.000 (0.000–0.000) | 0.000 |
| Serum.IgG | -0.062 | 0.026 | -2.346 | 0.940 (0.892–0.990) | 0.019 |

Table S5 Multivariate logistic regression

| Variables | beta | S.E | Z | OR[95%CI] | P_value |
| --- | --- | --- | --- | --- | --- |
| Ocular.symptoms | 1.186 | 0.397 | 2.991 | 3.275 (1.505–7.127) | 0.003 |
| Gastrointestinal.symptoms | 0.938 | 0.464 | 2.019 | 2.554 (1.028–6.348) | 0.043 |
| Water.swallowing.and.choking.difficulties | 1.131 | 0.547 | 2.066 | 3.097 (1.060–9.055) | 0.039 |
| Cerebral.lobes | -1.527 | 0.436 | -3.503 | 0.217 (0.092–0.510) | 0.000 |
| Centrum.semiovale | -1.397 | 0.561 | -2.488 | 0.247 (0.082–0.743) | 0.013 |
| Brainstem1 | 0.939 | 0.417 | 2.253 | 2.557 (1.130–5.786) | 0.024 |
| Cerebral.ganglia | 1.740 | 0.599 | 2.903 | 5.695 (1.760–18.431) | 0.004 |
| Spinal.cord | 1.990 | 0.435 | 4.573 | 7.319 (3.118–17.177) | 0.000 |
| APOA1 | 2.444 | 1.225 | 1.995 | 11.521 (1.044–127.173) | 0.046 |
| Ca | -4.051 | 1.841 | -2.200 | 0.017 (0.000–0.643) | 0.028 |
| Toxoplasma.gondii.IgM.Antibody | -3.502 | 0.752 | -4.655 | 0.030 (0.007–0.132) | 0.000 |
| CSF.IgG | -28.618 | 13.941 | -2.053 | 0.000 (0.000–0.274) | 0.040 |

Table S6 ROC curves of different model in training cohort

| Model.Type | AUC | Accuracy | F1.Value | Recall.rate | specificity | NPV | PPV | Brier.score | Threshold |
| --- | --- | --- | --- | --- | --- | --- | --- | --- | --- |
| LR | 0.936 | 0.878 | 0.667 | 0.900 | 0.875 | 0.982 | 0.529 | 0.065 | 0.154 |
| DT | 0.772 | 0.774 | 0.462 | 0.717 | 0.783 | 0.946 | 0.341 | 0.114 | 0.055 |
| RF | 0.919 | 0.912 | 0.702 | 0.767 | 0.935 | 0.962 | 0.648 | 0.073 | 0.283 |
| KNN | 0.877 | 0.819 | 0.565 | 0.867 | 0.812 | 0.975 | 0.419 | 0.074 | 0.183 |
| SVM | 0.929 | 0.907 | 0.709 | 0.833 | 0.919 | 0.972 | 0.617 | 0.066 | 0.197 |

TableS7 Comparison of AQP4-IgG-negative NMOSD versus Other nervous system autoimmune disorders Control group

| Comparison | Model | AUC | Accuracy | F1.Value | Recall.rate | specificity | NPV | PPV | Brier.score | Threshold |
| --- | --- | --- | --- | --- | --- | --- | --- | --- | --- | --- |
| AQP4-IgG-negative NMOSD vs MS | LR | 0.948921569 | 0.846153846 | 0.76635514 | 0.964705882 | 0.804166667 | 0.984693878 | 0.635658915 | 0.080881113 | 0.179193616 |
| AQP4-IgG-negative NMOSD vs GBS | LR | 0.992785794 | 0.956228956 | 0.928176796 | 0.988235294 | 0.943396226 | 0.995024876 | 0.875 | 0.027960797 | 0.196785672 |
| AQP4-IgG-negative NMOSD vs VE | LR | 0.998615917 | 0.994117647 | 0.99408284 | 0.988235294 | 1 | 0.988372093 | 1 | 0.012521216 | 0.625886891 |
| AQP4-IgG-negative NMOSD vs AE | LR | 1 | 1 | 1 | 1 | 1 | 1 | 1 | 3.93794E-21 | 0.5 |

Table S8 Trend test for AQP4 IgG negative NMOSD group

|  | neutrophil | | NLR | | NPR | |
| --- | --- | --- | --- | --- | --- | --- |
|  | *P* | chi-square | *P* | chi-square | *P* | chi-square |
| Clinlcal symptom | 0.009* | 0.037* | 0.034* | 0.109 | 0.139 | 0.234 |
| Imaging cumulative sites | 0.035* | 0.065 | 0.01* | 0.066 | 0.004* | 0.036* |
| Neuritis optica | 0.012* | 0.059 | 0.074 | 0.078 | 0.162 | 0.44 |
| Acute myelitis | 0.309 | 0.496 | 0.829 | 0.525 | 0.651 | 0.376 |
| Polar posterior region syndrome | 0.736 | 0.529 | 0.65 | 0.522 | 0.56 | 0.818 |
| Acute brainstem syndrome | 0.663 | 0.817 | 0.724 | 0.947 | 0.938 | 0.939 |
| Acute interencephalial syndrome | 0.184 | 0.306 | 0.021* | 0.027* | 0.193 | 0.32 |
| Cerebral syndrome | 0.087 | 0.129 | 0.302 | 0.74 | 0.316 | 0.031 |

Note: * is P <0.05

Logistic regression equation and probability calculation for AQP4-IgG-negative NMOSD

LP = −7.764

+1.712 × (Ocular symptoms)

+1.049 × (Gastrointestinal symptoms)

+1.253 × (Water swallowing and choking difficulties)

− 1.989 × (Cerebral lobes involvement)

− 1.213 × (Centrum semiovale involvement)

+1.648 × (Cerebral ganglia involvement)

+2.029 × (Spinal cord involvement)

+0.515 × (APOA1)

− 4.076 × (Serum calcium)

− 3.152 × (Toxoplasma gondii IgM antibody positivity)

− 23.360 × (CSF IgG level).

[Binary variables were coded as 1 when present and 0 when absent.]

The predicted probability was calculated as:**P = 1 / (1 + exp(−LP)).**

Using the Youden index derived from the training cohort, an optimal cutoff probability of 0.175 was identified. Patients with predicted probabilities ≥ 0.175 were classified as AQP4-IgG-negative NMOSD, whereas those below this threshold were classified as other CNS autoimmune diseases.

For example, consider a patient presenting with ocular symptoms and spinal cord involvement, without gastrointestinal symptoms, water swallowing and choking difficulties, cerebral lobar lesions, or centrum semiovale involvement. Cerebral ganglia involvement was absent. Laboratory evaluation showed an APOA1 level of 1.20 g/L, a serum calcium level of 2.15 mmol/L, negative Toxoplasma gondii IgM antibody, and a CSF IgG level of 0.045 g/L.

Using the final logistic regression model, the linear predictor (LP) was calculated as follows:

LP = −7.764

1.712 × 1 (ocular symptoms)

2.029 × 1 (spinal cord involvement)

0.515 × 1.20 (APOA1)

− 4.076 × 2.15 (serum calcium)

− 23.360 × 0.045 (CSF IgG)

which yielded an LP value of −1.23. The predicted probability was therefore calculated as:

P = 1 / (1 + exp(−LP)) = 1 / (1 + exp(1.23)) ≈ 0.226.

Based on the predefined cutoff probability of 0.175, this patient would be classified as AQP4-IgG-negative NMOSD.
